# The quest for a non-vector psyllid: Natural variation in acquisition and transmission of the huanglongbing pathogen ‘*Candidatus* Liberibacter asiaticus’ by Asian citrus psyllid isofemale lines

**DOI:** 10.1371/journal.pone.0195804

**Published:** 2018-04-13

**Authors:** El-Desouky Ammar, David G. Hall, Saeed Hosseinzadeh, Michelle Heck

**Affiliations:** 1 USDA-ARS, US Horticultural Research Laboratory, Fort Pierce, Florida, United States of America; 2 University of Florida, Lake Alfred & Fort Pierce, Florida, United States of America; 3 Boyce Thompson Institute, Ithaca, New York, United States of America; 4 USDA-ARS Emerging Pests and Pathogens Research Unit, Robert W. Holley Center for Agriculture and Health, Ithaca, New York, United States of America; 5 Plant Pathology and Plant Microbe Biology Section, School of Integrative Plant Science, Cornell University, Ithaca, New York, United States of America; University of Saskatchewan College of Agriculture and Bioresources, CANADA

## Abstract

Genetic variability in insect vectors is valuable to study vector competence determinants and to select non-vector populations that may help reduce the spread of vector-borne pathogens. We collected and tested vector competency of 15 isofemale lines of Asian citrus psyllid, *Diaphorina citri*, vector of ‘*Candidatus* Liberibacter asiaticus’ (*C*Las). *C*Las is associated with huanglongbing (citrus greening), the most serious citrus disease worldwide. *D*. *citri* adults were collected from orange jasmine (*Murraya paniculata*) hedges in Florida, and individual pairs (females and males) were caged on healthy *Murraya* plants for egg laying. The progeny from each pair that tested *C*Las-negative by qPCR were maintained on *Murraya* plants and considered an isofemale line. Six acquisition tests on *D*. *citri* adults that were reared as nymphs on *C*Las-infected citrus, from various generations of each line, were conducted to assess their acquisition rates (percentage of qPCR-positive adults). Three lines with mean acquisition rates of 28 to 32%, were classified as ‘good’ acquirers and three other lines were classified as ‘poor’ acquirers, with only 5 to 8% acquisition rates. All lines were further tested for their ability to inoculate *C*Las by confining *C*Las-exposed psyllids for one week onto healthy citrus leaves (6–10 adults/leaf/week), and testing the leaves for *C*Las by qPCR. Mean inoculation rates were 19 to 28% for the three good acquirer lines and 0 to 3% for the three poor acquirer lines. Statistical analyses indicated positive correlations between *C*Las acquisition and inoculation rates, as well as between *C*Las titer in the psyllids and *C*Las acquisition or inoculation rates. Phenotypic and molecular characterization of one of the good and one of the poor acquirer lines revealed differences between them in color morphs and hemocyanin expression, but not the composition of bacterial endosymbionts. Understanding the genetic architecture of *C*Las transmission will enable the development of new tools for combating this devastating citrus disease.

## Introduction

Huanglongbing (HLB), also known as citrus greening, is the most serious disease of citrus (reviewed in [1–5]), HLB symptoms include leaves with blotchy mottle, stunting, loss of root biomass, fruit drop, uneven fruit development, and ultimately tree death. HLB is found nearly worldwide where citrus is cultivated, including in the United States where it threatens the future of a multi-billion dollar industry in Florida, Texas and California. HLB affects all genotypes of *Citrus* and some other Rutaceae [2]. HLB in Asia, North and South America, Oceania and the Arabian Peninsula is strongly associated with plant infection by the Gram-negative bacterium ‘*Candidatus* Liberibacter asiaticus’ (*C*Las) [6, 7]. Two other Liberibacters are associated with HLB in other parts of the world; ‘*Ca*. L. americanus’ (*C*Lam) in Brazil and ‘*Ca*. L. africanus’ (*C*Laf) in Africa. The Asian citrus psyllid *Diaphorina citri* Kuwayama, 1908 (Hemiptera: Liviidae) is the natural vector of *C*Las and *C*Lam, whereas the Africa psyllid *Trioza erytreae* (Del Guercio, 1918) (Hemiptera: Triozidae) is the natural vector of *C*Laf. Experimentally, *D*. *citri* can transmit *C*Laf and *T*. *erytreae* can transmit *C*Las (reviewed in [7]).

*C*Las is transmitted in a circulative, propagative manner by *D*. *citri* [1, 8, 9]. Circulative, propagative transmission involves pathogen movement and replication in vector tissues prior to inoculation of a new host tree [10, 11]. Confocal microscopy and qPCR have been used to visualize or detect *C*Las in *D*. *citri* tissues. *C*Las was found in cells of the midgut, filter chamber, Malpighian tubules, hemolymph, salivary glands, muscle, fat body, and the reproductive organs [12–15]. As with other circulative, propagative pathogens, the process of *C*Las transmission can be broken down into three distinct phases: *C*Las acquisition into the vector, spread and replication in vector tissues, and inoculation of recipient host plants. During acquisition, *C*Las is ingested by *D*. *citri* during phloem feeding [16, 17] and acquired into the insect’s hemocoel, crossing the gut barrier [12–15]. During *C*Las acquisition, changes in transcriptome, proteome and protein interactions of *D*. *citri* immunity genes are observed [14, 15, 18, 19]; however, specific receptors for *C*Las in the vector are not known. *C*Las replicates in as of yet unidentified tissues in the insect vector and crosses into the salivary glands where it reaches high titer prior to inoculation [8, 12, 13]. During the inoculation process, psyllids deliver *C*Las into the phloem of a recipient host plant together with salivary secretions during feeding [20]. *D*. *citri* harbors at least three bacterial symbionts, ‘*Candidatus* Profftella armatura’, ‘*Candidatus* Carsonella ruddii’ and *Wolbachia pipientis* [21, 22]. The role of these symbionts in the biology of *D*. *citri* can be partially inferred from genome sequence and proteomics data [19, 21, 22]. Evidence shows that these symbionts have complex and possibly shared, coordinated metabolic and protein signaling networks with *C*Las [19, 23, 24] but a direct role for the bacterial symbionts in *C*Las acquisition and transmission has not been demonstrated.

Insect populations within a vector species vary in their ability to transmit plant and animal pathogens [25–29]. In 1925, Storey using *Maize streak virus* vectored by the leafhopper *Balclutha mbila*, (now known as *Cicadulina mbila* (Naude)), was the first to characterize individuals within an insect species that were “resistant” to pathogen acquisition despite multiple and extensive acquisition periods spent feeding on infected plant material [26]. Interestingly, Storey also reported that some progeny of resistant individuals were able to transmit in subsequent generations, which provided the first clues that such “resistance” was genetically encoded, a phenomenon he later observed in a different leafhopper species [30]. Since then, natural variation in the ability of insect vectors to transmit plant and animal pathogens has been demonstrated extensively in many vector species and has been used as a powerful tool to dissect the genetic and molecular mechanisms of vector competence and vector-pathogen interactions [27–29, 31–38].

Constructing a genetically engineered non-vector or inefficient vector psyllid for *C*Las (i.e., no evidence of *C*Las transmission by *D*. *citri*) was the original idea behind a $9 M dollar USDA NIFA and Florida Citrus Research and Development Foundation “NuPsyllid” project [39]. Although the project was extremely productive, the team was not able to generate a non-vector psyllid due to the lack of tools available for psyllid transformation. Finding a poor or inefficient vector in nature and studying vector competence determinants in *D*. *citri* will greatly enhance our ability to devise novel control methods for this devastating HLB citrus disease in the USA and elsewhere. It has been previously reported that substantial variability exists in the infection rate of in *D*. *citri* field populations [40, 41], which can be attributed to genetics and/or environmental or host plant factors. In this work, we established *D*. *citri* isofemale lines to investigate the genetic variability in *C*Las transmission in natural *D*. *citri* populations. In his 1932 paper, Storey referred to the ability of a leafhopper to transmit plant viruses as “activity” and the absence of such activity to transmit “inactivity.” Our understanding of pathogen transmission by insects has enabled us to break down transmission “activity” into distinct processes, which we characterized here. Specifically, we characterize the phenotypes of the isofemale lines for 1) *C*Las acquisition, which here encompasses both acquisition and spread/replication in insect tissues, and 2) inoculation, which is the ability of the insect to deliver the pathogen during feeding onto a recipient, susceptible host plant. It is also important to clarify that, *C*Las-exposed psyllids are those that fed on *C*Las-infected plants, but only the individuals which have acquired *C*Las to detectable levels by qPCR are considered *C*Las-infected, and that some *C*Las-infected individuals may not be capable of transmitting/inoculating *C*Las into citrus.

## Results

### Geographic distribution of founder females

Live *D*. *citri* adults were collected in Florida from several locations in the cities of Fort Pierce, Port St. Lucie, Homestead and Fort Myers between 7/11/2014 and 10/28/2015 (S1 Table). Males and females were collected from hedges of orange jasmine, *Murraya paniculata* (L.), using either a mouth aspirator or a sweep net, for the latter then a mouth aspirator to collect psyllids from the net into glass collection tubes. Orange jasmine hedges in residential areas (away from commercial citrus groves) were chosen for *D*. *citri* collection to minimize the risk of collecting *C*Las-infected psyllids because, while orange jasmine plants are known to be good hosts for *D*. *citri*, they are much less favorable to *C*Las infection and multiplication [42–44].

### Isofemale lines of *D*. *citri* vary in their ability to acquire *C*Las, and the acquisition phenotype is heritable for several generations

**Fig 1:**
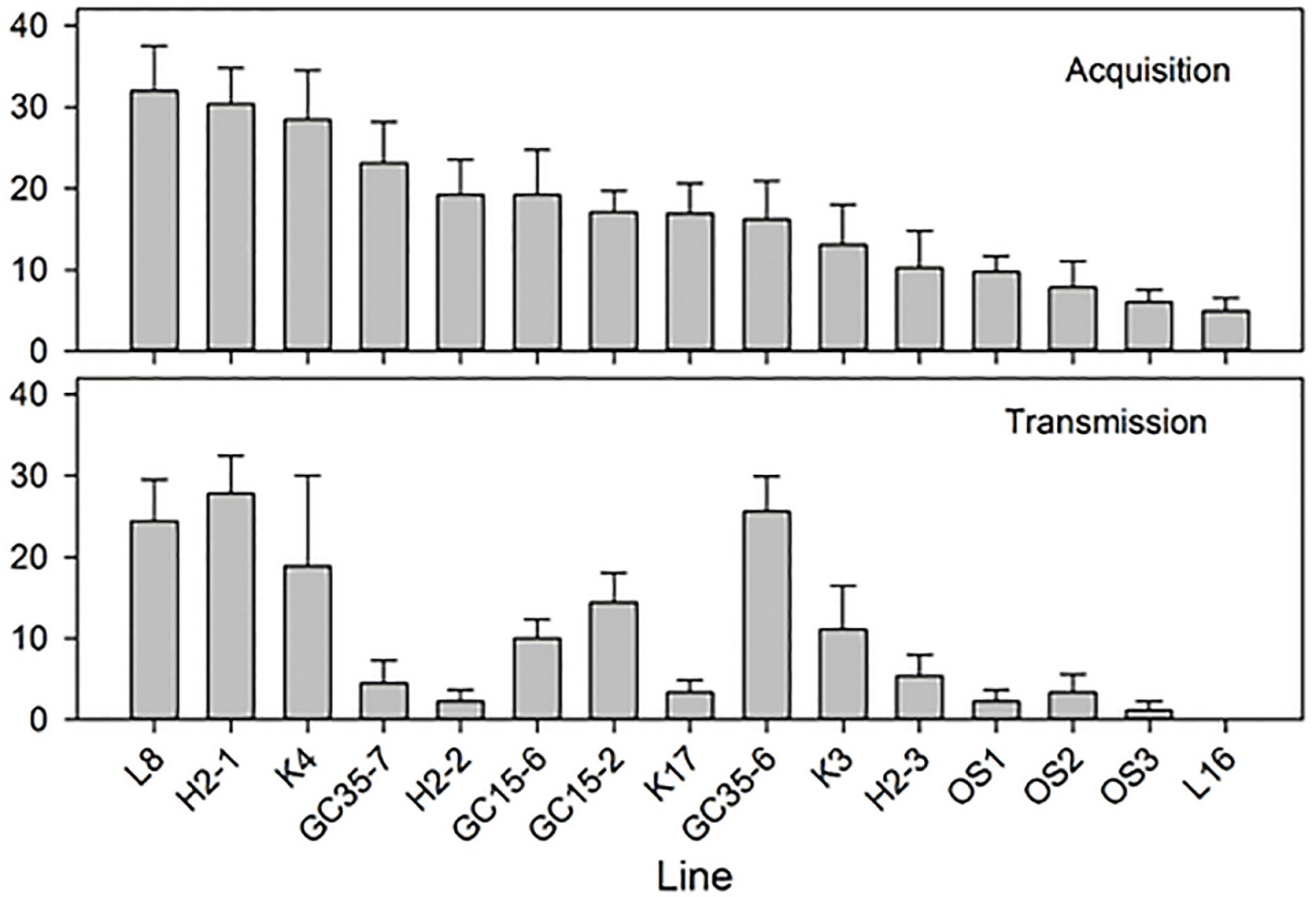
Mean ‘*Candidatus* Liberibacter asiaticus’ (*C*Las) acquisition and transmission rates of *Diaphorina citri* isofemale lines collected from throughout the state of Florida. Lines are organized according to their *C*Las acquisition rate. Shown are means ± standard error of the means of psyllids from 15 tested isofemale lines. Corresponding inoculation/transmission rate is shown below the acquisition rate for each line.

Each isofemale line was tested for *C*Las acquisition from infected (bud-inoculated) rough lemon plants six times over the span of several (4–27) generations by qPCR using *C*Las-specific primers. A highly significant difference (*P*<0.0001) was found among the 15 isofemale lines tested with respect to mean acquisition rates, i.e., percentage of *C*Las-exposed psyllids that tested positive for *C*Las in six acquisition tests (Tables 1 and 2, S2 Table, Fig 1). In a broad sense, there were three acquisition phenotypes observed: high, medium, and low. The best three ‘acquirer’ lines were L8, H2-1 and K4, with mean acquisition rates of 28–32%. The three poorest acquirer lines were L16, OS3 and OS2, with 5–8% mean acquisition rates (Table 1). The remaining lines had acquisition rates falling in between the best and worst acquirer lines. There were also highly significant differences among lines in the mean cycle threshold (Ct) value of *C*Las in infected psyllids, which ranged between 31.01 (higher *C*Las titer) in Line L8 (the best acquirer line) and 35.30 (lower *C*Las titer) in Line OS1 that also had a low acquisition rate of 10% (Tables 1 and 2). In the healthy control (*C*Las-unexposed) psyllids, 0.18% (3/1687) tested *C*Las positive in qPCR tests (compared to 5–32% in the *C*Las-exposed psyllids), which we attributed to possible mislabeling or contamination of the qPCR samples.

**Table 1:** ‘*Candidatus* Liberibacter asiaticus’ (*C*Las) acquisition rates and Ct values of *Diaphorina citri* isofemale lines.

No	Line^1^	Generation^2^	Mean% acquisition rate ^3^^,^^4^	Mean psyllid Ct value^3^^,^^4^
**1**	L8	24	31.99a	31.01dc
**2**	H2-1	6	30.39a	32.49abcd
**3**	K4	27	28.42ab	32.73abcd
**4**	GC35-7	5	23.11abc	31.03d
**5**	H2-2	7	19.22abcde	34.18ab
**6**	GC15-6	6	19.18abcd	31.49bcd
**7**	GC15-2	6	17.09abcde	33.40abcd
**8**	K17	14	16.93abcd	34.21ab
**9**	GC35-6	6	16.17bcdef	33.21abcd
**10**	K3	19	13.12cdefg	34.28abc
**11**	H2-3	5	10.21efg	31.48bcd
**12**	OS1	4	09.73defg	35.30a
**13**	OS2	10	07.80fg	35.17a
**14**	OS3	5	06.03g	34.55a
**15**	L16	20	04.98g	34.39a

Shown in column four are the mean percentages of psyllids that acquired *C*Las from infected citrus and in column five are the mean Ct values of these psyllids in qPCR tests.

^**1**^ Lines are arranged in descending order according to mean acquisition rate.

^**2**^ Estimated no. of generations between the first and last acquisition tests; generation time was estimated to be 3–4 weeks.

^**3**^ Six acquisition tests/line with ca. 27–158 psyllid adults/test; tested psyllids had been fed as nymphs on *C*Las -infected lemon plants for one or more generations.

^4^ Means followed by different letters are significantly different at *P* <0.05 (nonparametric analyses of variance on rankings, raw mean percentages and Ct values presented).

**Table 2:** Type III tests on variability among isofemale lines for data presented in Table 1.

Parameter	Num DF	Den DF	F value	*P* value
Percent acquisition by psyllids	14	70	5.0	<0.0001
Psyllid Ct value	14	67	2.1	0.02

Two of the best acquirer lines (L8 and K4) were tested for *C*Las- acquisition through a period spanning 24–27 generations, and two of the poorest acquirers (OS2 and L16) were tested through a period spanning 10–20 generations (Table 1, S2 Table). No correlation was found between the generation tested and the acquisition rates in all tested lines except for line GC15-2 (tested for 6 generations) which had a significantly positive slope between these two variables (P <0.05, S3 Table, and S1 and S2 Figs); i.e. the acquisition rates for this line were higher in later generations compared to earlier ones. However, this was not true for the other 14 lines tested, some over a period of up to 27 generations (Table 1, S2 and S3 Tables).

### *C*Las acquisition rate and titer in the isofemale lines are positively correlated

**Fig 2:**
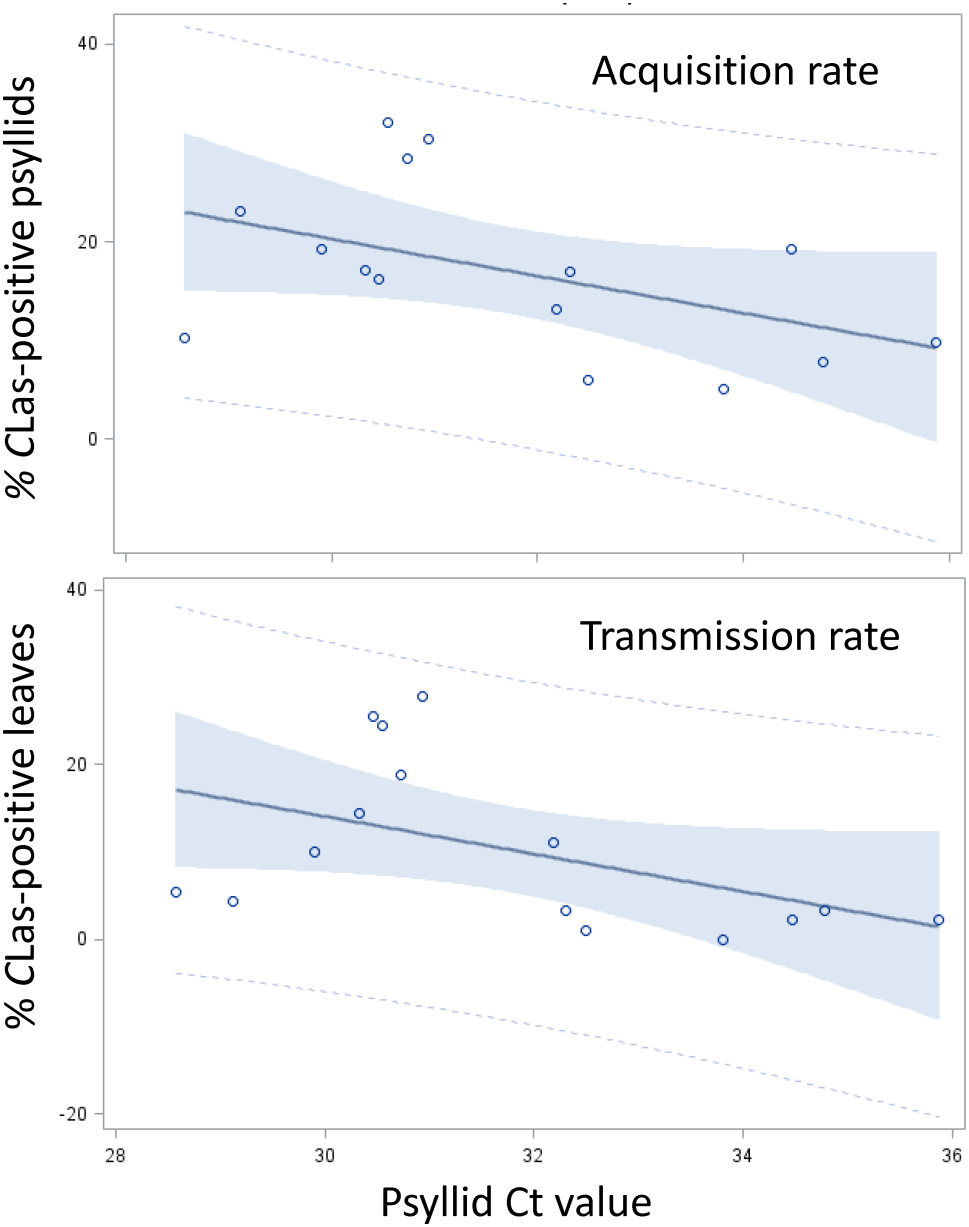
Linear regression between ‘*Candidatus* L. asiaticus’ (*C*Las) Ct values in *Diaphorina citri* isofemale lines and *C*Las acquisition and transmission rates. Correlation analysis between mean psyllid Ct values in qPCR tests, *C*Las-acquisition rate (A) and *C*Las-transmission rates (B) by psyllids of the isofemale lines tested. Shaded areas indicate 95% confidence limits.

Average Ct values for the tested isofemale lines ranged from 30.01 to 35.30 (Table 1). Four lines, L8, K4, GC35-6, and K3, showed the lowest average Ct values. Line OS1 showed the highest average Ct value. A significant (*P* = 0.012), negative correlation was found between acquisition rates and psyllid Ct values among the 15 tested lines (Table 3, Fig 2A). This indicates a positive correlation between percent acquisition and *C*Las titer in psyllids, as in qPCR lower Ct values are associated with higher *C*Las titers and vice versa. Two lines, K3 and GC35-6, had Ct values lower than other isofemale lines with similar acquisition rates. One line, H2-2, had a higher Ct value that other isofemale lines with similar acquisition rates. The combined *C*Las acquisition and Ct value data for these three lines suggests that *C*Las acquisition and regulation of *C*Las titer in the insect may be two distinct traits controlled by overlapping but not identical genes.

**Table 3:** Pearson correlation coefficients and linear regression analyses on the acquisition and inoculation/transmission data of isofemale lines.

Independent variable (X)	Dependent variable (Y)	*R*	Intercept	Slope	Slope *t*-value	N	*P*
Acquisition rate	Psyllid Ct value	-0.63	140.6	-3.730	-2.93	15	**0.012**
Psyllid Ct value	Acquisition rate	-0.63	35.0	-0.107	-2.93	15	**0.012**
Psyllid Ct value	Transmission rate	-0.68	139.71	-3.99	-3.03	15	**0.005**
Acquisition rate	Transmission rate	0.66	-0.3	0.668	3.14	15	**0.008**
Acquisition rate	Leaf Ct value	-0.03	35.9	-0.007	-0.12	14	0.906
Transmission rate	Leaf Ct value	0.13	35.5	0.026	0.47	14	0.648
Psyllid Ct value	Leaf Ct value	0.34	26.6	0.289	1.25	14	0.234

### Sex and gut clearing of the psyllids have no effect on *C*Las acquisition but impact titer levels in *D*. *citri*

Hemipteran vectors can ingest many plant pathogens that they do not necessarily acquire or transmit (reviewed in [36]). This raised the concern that the acquisition and *C*Las titer calculations using our procedures during the above experiments possibly reflected ingested, rather than truly acquired, *C*Las bacteria from the plant. Using a positive control lab colony of *D*. *citri*, which consists of mixed, non-isofemale line psyllids that have been continuously reared on *C*Las-infected citron (*Citrus medica* L.), we tested whether gut clearing of the psyllids by feeding them for 24 h on healthy citrus leaves would have an effect on the percentage of insects that test *C*Las-positive or their Ct values. No significant differences were detected in the rate of *C*Las acquisition between gut-cleared and non-cleared psyllids (Table 4). However, Ct values were slightly but significantly higher in the psyllids that were exposed to the healthy citrus leaves for gut clearing (P<0.008, Table 4). This might have been due to possible *C*Las multiplication in the psyllids that were placed on healthy leaves for 24 h. Sex of the insect had no effect on the rate of *C*Las detection, showing that males and females do not differ in their acquisition rates. However, Ct values were significantly lower (indicating higher *C*Las titer) in infected females compared to infected males (P<0.001, Table 4), which supports field observations on the rate of infected *D*. *citri* in Florida [41]. Strikingly, *D*. *citri* colonies continuously reared on *C*Las-infected citron (Table 4) had significantly higher acquisition rates and higher titer as compared to any of the isofemale lines tested (Table 1).

**Table 4:** Effects of gut-clearing and gender on ‘*Candidatus* Liberibacter asiaticus’-acquisition rates and Ct value in an infected positive-control *Diaphorina citri* colony (non-isofemale line).

Parameter	Category	Infected/total number	Percent infected	Mean Ct value	*P*	
					Ratio infected psyllids^1^	Ct value^2^
Gut clearing	Gut- cleared	58/60	96.7	28.60±0.85	0.50	0.008
	Not- cleared	59/60	98.3	30.13±0.92		
Gender	Male	67/68	98.5	30.20±0.68	0.40	0.001
	Female	50/52	96.2	28.26±1.12		

Gut clearing had no impact on the percentage of insects that test positive but did have an impact on Ct values insects where their guts were cleared on healthy citrus plants.

^1^ Chi-square analysis.

^2^ *t* test.

### Isofemale lines vary in their *C*Las transmission rates

**Fig 3:**
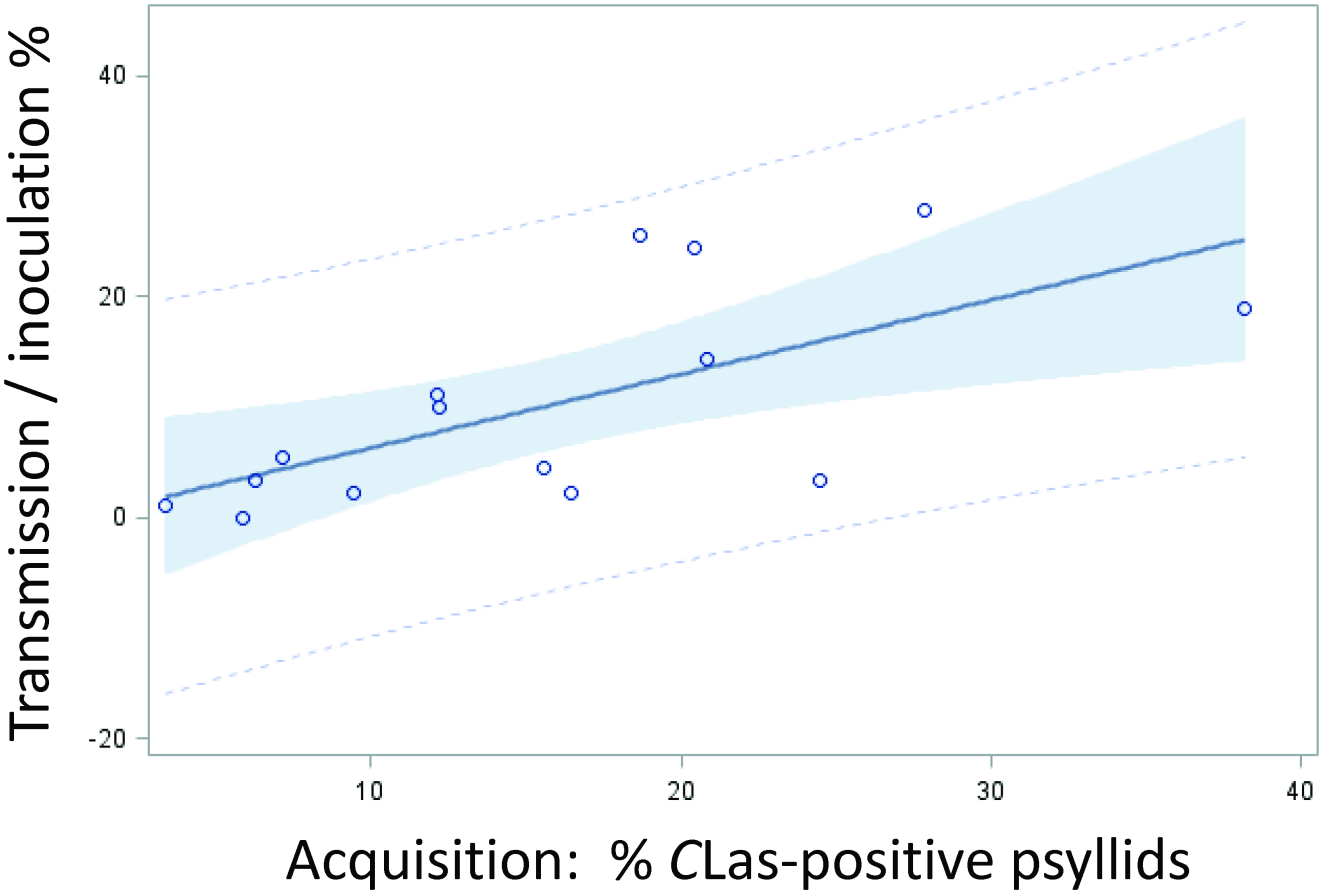
Linear regression (correlation analysis) between ‘*Candidatus* Liberibacter asiaticus’ (*C*Las) -acquisition and *C*Las-transmission rates by *Diaphorina* isofemale lines. Shaded area indicates 95% confidence limits.

We tested the ability of *C*Las-exposed adults from each isofemale line, that have been reared for one or more generations on infected citrus, to transmit/inoculate *C*Las into healthy citrus using our excised leaf assay method previously described [45]. In this paper, we use the term “transmission rate”, which is more widely used in the literature, to indicate ‘the inoculation rate’ that we obtained in our inoculativity tests. *C*Las transmission by each isofemale line was tested three times by inoculating young excised sweet orange leaves for two weeks with *C*Las-exposed psyllids (6–10 adults/leaf/week, two sets of healthy leaves consecutively inoculated with the same psyllids, S2 Table). Our results show that tested lines can be classified as to having different transmission phenotype rates (Table 5), ranging from the highest average transmission rate of 27.8% (line H2-1) to 0% (line L16). Highly significant differences in the transmission rate (% *C*Las-positive inoculated leaves) were found between various lines (Table 6, *P* = 0.0001). These tests showed that the three best acquirer lines (L8, H2-1 and K4), in addition to line GC35-6, were also the best *C*Las inoculation lines, with inoculation rates of 19 to 28% reflecting relatively high acquisition and inoculation rates by populations of these psyllids (Fig 1). Additionally, the poorest acquirer lines (L16, OS1, OS2, OS3), in addition to Line H2-2, were also the poorest inoculation lines, with inoculation rates of 0 to 2% reflecting poor acquisition and inoculation rates by populations of these psyllids (Tables 1 and 5, Fig 1). A positive correlation between acquisition and transmission rates was also highly significant (*P* = 0.008, Table 3, Fig 3). A negative correlation was found between transmission rate and mean psyllid Ct value of each line, which indicates a positive correlation with *C*Las titer (Table 3, Fig 2B). However, there was no significant correlation between acquisition or inoculation rate with mean Ct values of infected leaves (Table 3). It is interesting to note that the mean Ct values of leaves inoculated by insects from line GC35-7 were considerably lower, indicating higher *C*Las titer, as compared to leaves inoculated by all other isofemale lines used in these tests (Table 5).

**Table 5:** ‘*Candidatus* Liberibacter asiaticus’ (*C*Las) inoculation/transmission rates by *Diaphorina citri* isofemale lines.

No	Line^1^	Mean % transmission rate ^2^^,^^3^	Mean leaf Ct value^2^^,^^3^
**1**	H2-1	27.78a	36.19a
**2**	GC35-6	25.56ab	36.01a
**3**	L8	24.44ab	35.72a
**4**	K4	18.89cde	36.04a
**5**	GC15-2	14.44abc	37.08a
**6**	K3	11.11cde	36.27a
**7**	GC15-6	10.00bcd	35.47a
**8**	H2-3	5.36def	37.35a
**9**	GC35-7	4.44ef	29.81a
**10**	OS2	3.33ef	36.41a
**11**	K17	3.33def	35.03a
**12**	OS1	2.22ef	36.78a
**13**	H2-2	2.22ef	37.13a
**14**	OS3	1.11f	35.38a
**15**	L16	0.00f	-^4^

Mean percentage of *C*Las-positive sweet orange leaves is shown in the third column, and their mean Ct values in the fourth column, following an inoculation access period of 7 days with *C*Las-exposed psyllids (6–10 psyllids/leaf).

^**1**^ Lines arranged in descending order according to mean transmission rate.

^**2**^ Three transmission tests/line, 15 excised leaves/test/week for 2 consecutive weeks (90 leaves total), with 6–10 psyllids/leaf/week; inoculating psyllids had been fed as nymphs on *C*Las-infected lemon plants for one or more generations.

^3^ Means followed by different letters are significantly different at *P* <0.05 (nonparametric analyses of variance on rankings, raw mean percentages and Ct values presented).

^4^ None of the tested leaves in Line L16 were *C*Las-positive by qPCR.

**Table 6:** Type III tests on variability among isofemale lines for data presented in Table 5.

Parameters	Num DF	Den DF	F value	P
% psyllids infected	14	73	19.1	<0.0001
Psyllid ct value	14	26	4.7	0.0004
% transmission	14	73	6.6	<0.0001
Mean leaf Ct value	13	33	1.26	0.28

### Phenotypic and molecular characterization reveals differences between L8 and L16 in the ratio of color morphs and hemocyanin expression

**Fig 4:**
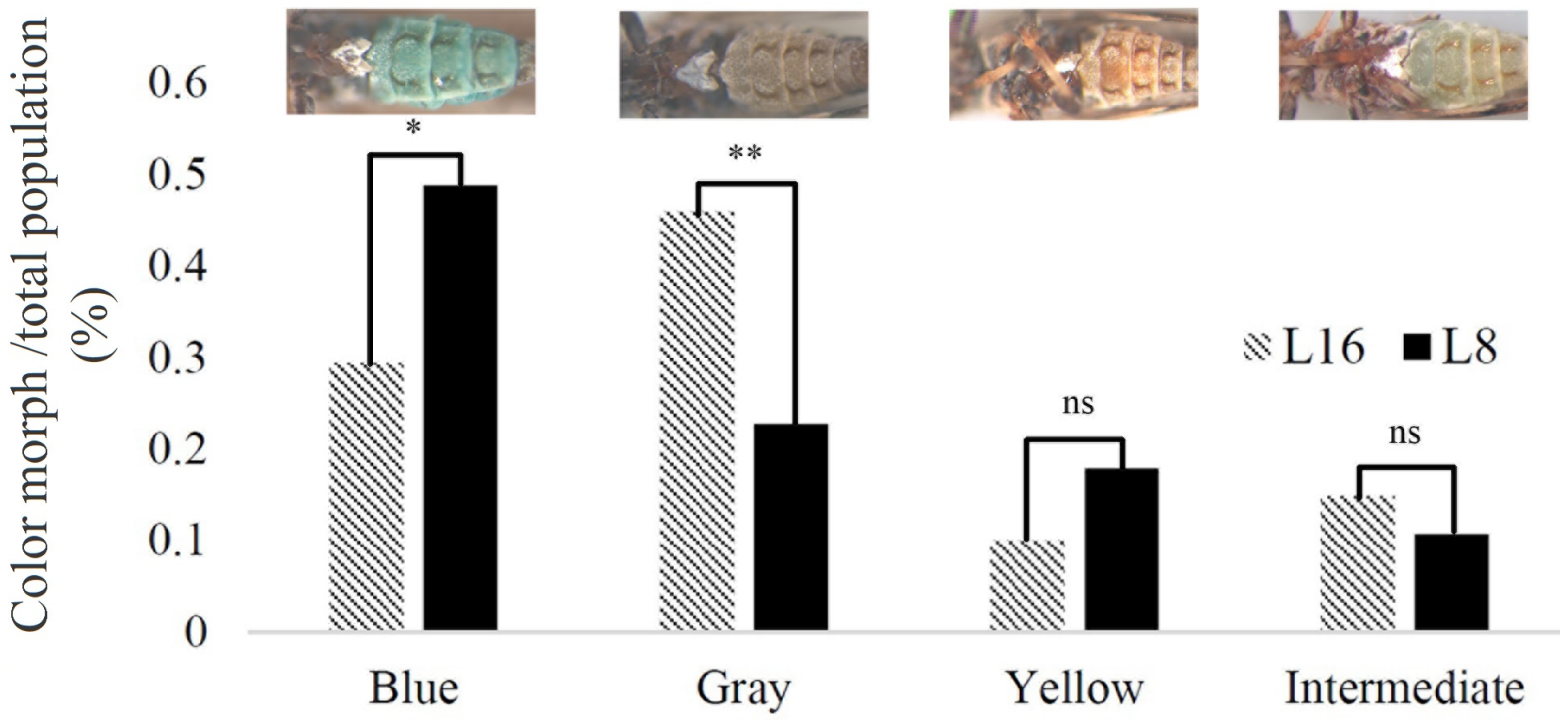
Isofemale lines vary in proportion of color morphs. Percentages of four color morphs between the psyllids of two isofemale lines (L8 and L16) show significant differences in the blue and gray color morphs but not yellow and intermediate.

**Fig 5:**
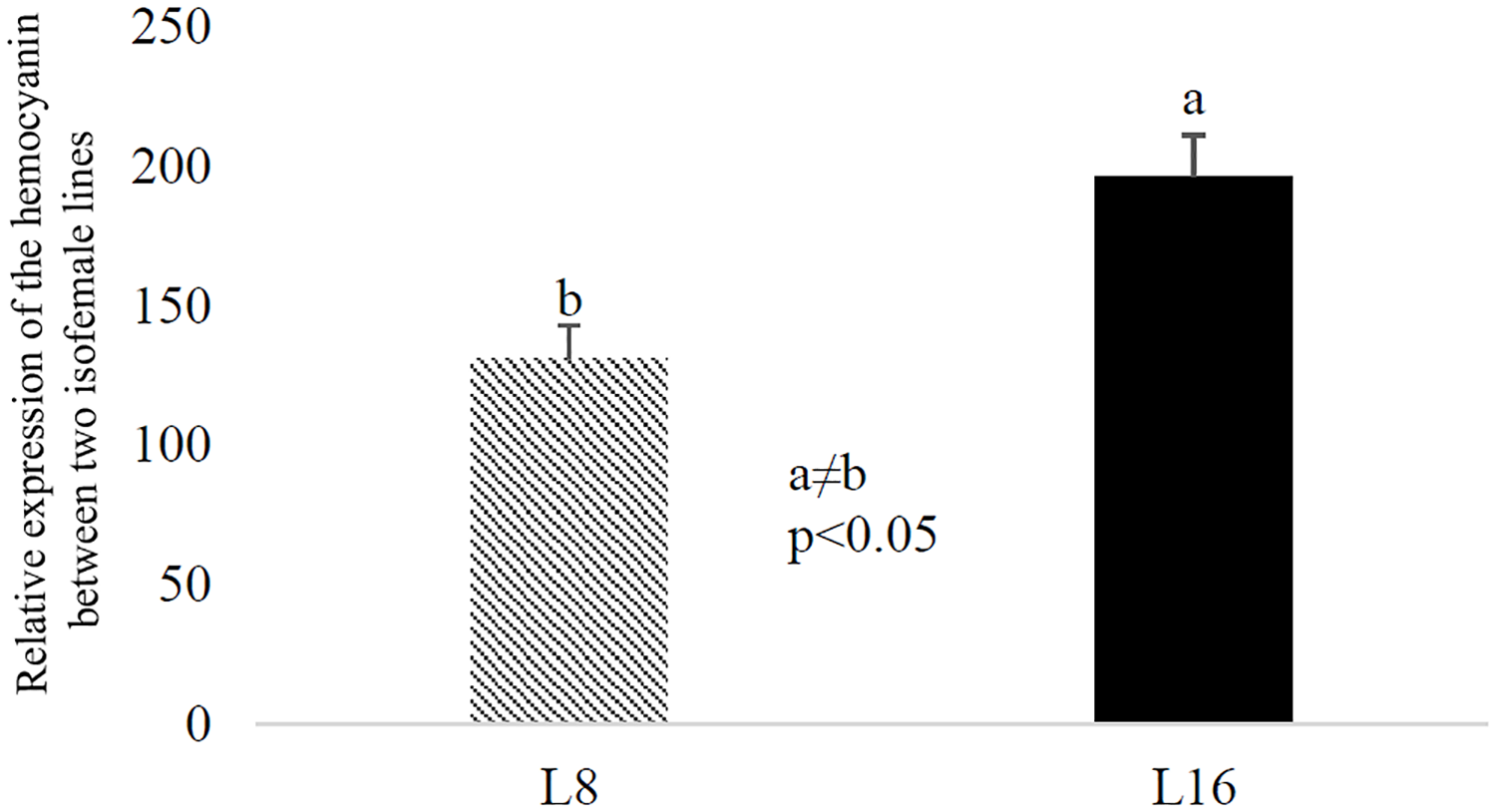
Expression of *Diaphorina citri* hemocyanin-1 gene quantified by q-RT-PCR in two isofemale lines. Normalized, relative expression of *D*. *citri* hemocyanin-1 gene using q-RT-PCR in L8 and L16 using hemocyanin-1 gene specific primers. The gene is expressed at higher levels in L16 as compared to L8.

**Fig 6:**
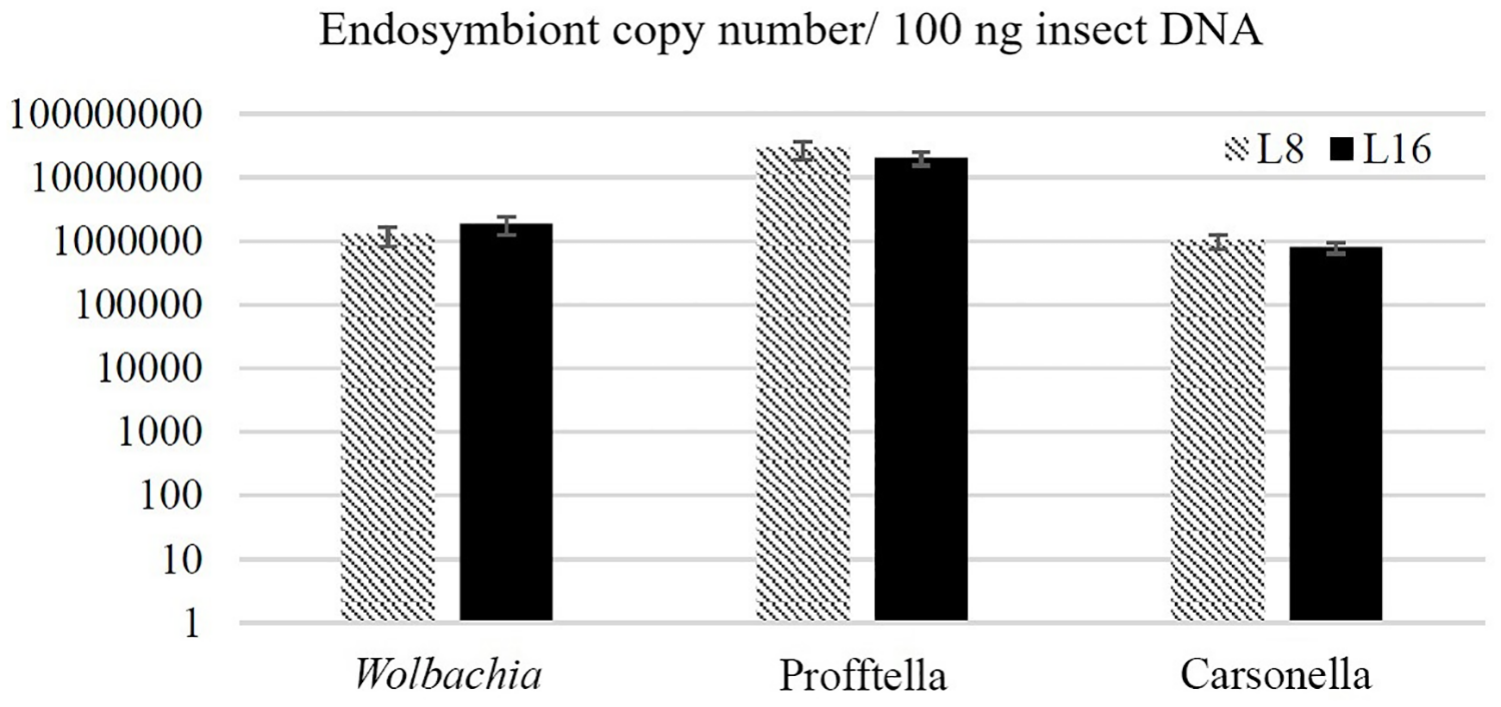
Quantification of the three *Diaphorina citri* bacterial symbionts using qPCR in two isofemale lines. The population of endosymbionts *Wolbachia pipientis*, ‘*Candidatus* Proftella armatura’ and ‘*Candidatus* Carsonella rudii’ show no differences in abundance between *D*. *citri* isofemale lines L8 and L16.

Phenotypic variation in *D*. *citri* has been described in color morphology [46, 47] and bacterial endosymbiont density [48]. Thus, we hypothesized that the isofemale lines may differ in characteristics other than *C*Las acquisition and inoculation. We characterized morphological, endosymbiont, and gene expression variation between the best and poorest acquirer lines, L8 and L16. There was no difference in size between L8 and L16 individuals of either sex (data not shown). *D*. *citri* populations generally contain three broadly different abdominal color morphs [46]. Careful examination of our isofemale lines further inspired four color morph categories, blue, grey, yellow and intermediate. Intermediate insects had a pale blue tinge to their abdomens but were not as blue as those classified as blue (Fig 4). Insects in L8 and L16 showed variation in the proportions of the different color morphs (Fig 4). In general, the brown L16 were lighter in color than brown L8 individuals. Additionally, L8 had significantly more blue *D*. *citri*, whereas L16 had significantly more grey (Fig 4). No differences in the proportion of yellow and intermediate color morphs were found between L8 and L16 (Fig 4). Hemocyanin is a copper-binding protein with a distinct blue color [18]. Consistent with its known role as an immune response protein in insects, it is one of the most highly expressed proteins in the *D*. *citri* proteome when the insects are reared on *C*Las -infected trees [18]. We measured hemocyanin transcript expression in L8 and L16, and L16 expressed significantly more hemocyanin as compared to L8 (Fig 5, P<0.05). No clear differences in endosymbiont titer were observed between *D*. *citri* from the L8 and L16 lines reared on healthy citrus plants (Fig 6).

## Discussion

We showed that natural variation in *C*Las acquisition and inoculation/ transmission exists among *D*. *citri* populations of the isofemale lines tested from Florida. In our work, we phenotyped adults for *C*Las acquisition/infection rates and used *C*Las-exposed adults in inoculation experiments. The variation in *C*Las transmission (acquisition and/or inoculation) ability observed among the isofemale lines may result from a number of factors, acting alone or in various combinations. One possibility is that these variations could be due to genetic variation in the ability of nymphs to acquire *C*Las from infected plants. For adults to efficiently transmit *C*Las, the bacterium must be acquired during the nymphal stage [8, 9]. Two separate studies showed that no adult *D*. *citri* individuals that acquired *C*Las only during the adult stage were able to inoculate citrus seedlings [8, 9]. Nymphs acquire *C*Las more efficiently than adults [8, 9, 49], and *C*Las replicates faster and to higher levels in nymphs than in adults [9]. Gut and salivary gland barriers to *C*Las acquisition and/or transmission by *D*. *citri* have been suggested earlier [12, 13]. These barriers may be more permissive in the nymphs compared to adults, which can explain this stark difference in both acquisition and transmission between *D*. *citri* nymphs and adults. Morphological, physiological and feeding behavior differences between nymph and adult *D*. *citri* may also contribute to differences in *C*Las acquisition by nymphs and adults. Midgut epithelial cells in nymphs [50], which are the first barrier to *C*Las acquisition into *D*. *citri* tissues, are resistant to the induction of apoptosis (programmed cell death) that is observed extensively in the adult midgut [15, 50]. There may be a correlation between *C*Las acquisition phenotype and color morphology. Isofemale lines L8 and L16 varied most greatly in their ability to acquire and transmit *C*Las. They also varied in their proportion of color morphs produced, with L8 producing a greater proportion of blues as compared to L16, which produced more grey. The grey color morph is usually the least abundant in field populations in Florida [46]. Further experiments would be necessary to correlate color morphology to *C*Las acquisition and transmission phenotypes in *D*. *citri*.

Genetic variation in the ability of the insect to suppress *C*Las replication may also explain the variation in *C*Las transmission among isofemale lines. Significant differences in average *C*Las titer were observed among different lines, with some lines developing lower or higher titers than other lines that acquired at a similar rate. A quantitative proteomics analysis showed that nymphs of *D*. *citri* express a suite of proteins that may be involved in *C*Las acquisition and replication at higher or lower levels as compared to adult insects. For example, mucins (bacterial adhesion proteins) are more highly expressed in nymphs. In contrast, transferrin proteins, predicted to function in the inset immune response by sequestering iron from bacterial pathogens, were below the limit of detection in nymphs and expressed at very high levels in adults [18]. Variation in the expression of these proteins, and others, may exist among the isofemale lines.

Our data provide evidence in support of the hypothesis that distinct genes in the *D*. *citri* regulate *C*Las acquisition and inoculation/transmission abilities. As such, acquisition and replication may depend on two distinct sets of barriers that *C*Las must overcome for successful transmission into a new host plant. Although we observed a positive correlation between rates of acquisition and inoculation, at least two of the lines defied these trends. Intriguingly, both of these lines were derived from *C*Las-positive lab colonies that had been established over ten years ago. Isofemale line GC35-7 showed a good *C*Las acquisition phenotype and a poor inoculation phenotype. In contrast, GC35-6 showed an intermediate acquisition phenotype and a good inoculation phenotype. In aphids, distinct aphid genes and proteins regulate barriers to transmission of circulative viruses in the *Luteoviridae* [28, 29, 32, 51, 52], although unlike *C*Las the circulative viruses are non-propagative in their aphid vectors [36]. Not all insect vectors have multiple genes encoding for vectoring ability. The vectoring ability of *Frankliniella occidentalis* Pergan de, 1895 for *Tomato spotted wilt virus* is due to a single recessive allele [35]. In *F*. *occidentalis*, allele frequencies were different in males and females. However, unlike the aphids and thrips, which can reproduce parthenogenically, the obligate sexual reproduction in *D*. *citri* would make genetic studies on multiple recessive alleles regulating vector competence more complicated.

We did not test for differences in acquisition between males and females in our isofemale populations, but our results from the positive control colony (a non-isofemale line colony reared on infected citron for many generations) showed no significant differences in *C*Las acquisition between the two sexes (Table 4). Although males can sexually transmit *C*Las to females at very low rates (<4%) [53], sexual transmission of *C*Las is not likely to be a confounding factor in the acquisition and transmission phenotypes we characterized from because we started our experiments with nymphs reared on healthy trees. Females from these positive control colonies developed higher titers of *C*Las as compared to males. In the alimentary canal, *C*Las was shown to induce higher levels of oxidative stress in males as compared to females [50]. The increase in production of reactive oxygen species in the male midgut may act to negatively regulate *C*Las titers. When analyzing the isofemale lines for acquisition and transmission, we selected insects at random and did not control for the sex of the insect, but the sex of the insect should be taken into account in future studies.

Previous evidence strongly supports the idea that *C*Las replicates in *D*. *citri*, especially in nymphs [9]. In this framework *D*. *citri* is considered a susceptible host for *C*Las, and we hypothesize that variation in the insect’s immune response may play a role in the ability of the insect to harbor, support multiplication and successfully transmit *C*Las and hence, in the variation we observed among the isofemale lines. Annotation of the *D*. *citri* genome revealed a reduced innate immune system [54], which as in their other hemipteran relatives, is thought to have evolved to support the relationship between the insect and a diverse community of microbial endosymbiotic partners [55–57]. In spite of the reduced repertoire of classical immunity genes, proteomic analysis of adult *D*. *citri* reared on *C*Las -infected or healthy trees showed that *C*Las induces changes in the expression of proteins involved in ATP synthesis, the cytoskeleton, endocytosis, and immunity [14, 18, 19]. One such example is the *D*. *citri* protein hemocyanin-1. Hemocyanin-1 was the most highly expressed proteins in *C*Las-exposed adult *D*. *citri* [18]. In that study, a quantitative proteomic analysis on *C*Las-exposed insects was conducted using high resolution mass spectrometry. Infection rates of the biological replicate pools of insects ranged from 53% to 90%. Thus, since not all insects in each *C*Las-exposed pool were infected, it was not known whether hemocyanin-1 expression was upregulated in non-infected or infected insects, or possibly both (as would be expected if the gene were induced by *C*Las infection in the host plant, and not the insect). Here, the poor vector line L16 showed a higher level of hemocyanin-1 expression as compared to L8, the good vector line. If hemocyanin does play a role in the insect immune response to *C*Las it may be that higher levels of hemocyanin-1 suppress *C*Las replication in the insect.

*C*Las was shown to induce apoptosis in the midgut of adult *D*. *citri* [15, 50], which is a likely site of *C*Las acquisition and replication. The gut of *C*Las-exposed adults shows proteomic signatures of programmed cell death, including a concerted down-regulation of proteins involved in mitochondrial function and the TCA cycle [14]. The genetic factors controlling this response in the adults may vary among the *D*. *citri* isofemale lines. The rates of inoculation/transmission in our study for the three best vector lines ranged from 24 to 28%. In previous studies, much lower percentages of *D*. *citri*, ranging from 1.3–12.2%, have been reported to inoculate citrus seedlings although much higher rates (reaching 90%) of the insects may test positive for *C*Las [9]. We believe that the number of insects used in the inoculation assays in our study can explain this discrepancy. Here, we used 6–10 insects per leaf, whereas 1–5 insects per plant or leaf were used in most previous studies. Evidence from the literature supports this idea. Pelz-Stelinski et al. reported that successful transmission of *C*Las by individual psyllids ranges from 4 to 10%, whereas groups of 100 or more insects transmit the pathogen 88% of the time [49]. For aphids and whiteflies that transmit circulative plant viruses, the number of insects per test plant is a critical experimental parameter [58–60]. Three previous studies compared *C*Las inoculation by *D*. *citri* into excised citrus leaves with that of inoculating whole plants/seedlings. Inoculation rates using these two methods were comparable in two studies [45, 61], but the whole plant method gave higher inoculation rates in a third study [9]. However, using excised leaves, rather than whole plants for inoculation saves considerable time, effort and material, especially when extensive tests are to be undertaken and the time factor is crucial as is the case with *C*Las and/or HLB [45, 61].

The acquisition rates obtained from our best three acquirer lines (28 to 32%) (Table 1) were considerably lower than the acquisition rate we observed, using the same qPCR and other procedures, from a non-isofemale colony that we reared on infected citron for many generations (Table 4). The reasons for this are unclear, but we hypothesize that this might be due to host plant effect on the psyllids ability to acquire *C*Las from diseased citrus. The parents of our isofemale lines were collected from *Murraya* (orange jasmine) hedges to minimize the chances of starting with *C*Las-infected parents from the field, because we know that *Murraya*, although a good host for *D*. *citri*, is much less susceptible to *C*Las than citrus [42, 43]. For the same reason, and to minimize *C*Las contamination in our isofemale lines, they were maintained on healthy *Murraya* plants for several generations before they were tested for *C*Las acquisition and transmission by feeding them on *C*Las-infected citrus (rough lemon). *Murraya* may have traits that select for psyllids of lower vector competency. We are currently investigating this possibility, and doing further acquisition and transmission tests on our isofemale lines to see if the phenotypes we found so far are stable or are still heterogeneous with regard to *C*Las acquisition and transmission abilities. In aphids, the host plant on which the aphids are reared can have a measureable impact on the aphid’s ability to transmit plant viruses [62–64]. Alternatively, since these non-isofemale line *D*. *citri* were reared on *C*Las infected plants for many generations, it is possible that selection has favored the proliferation of a highly efficient *D*. *citri* transmitter genotype. A previous study by Pelz-Stelinski and Killiny showed that *D*. *citri* harboring *C*Las were more fecund than their uninfected counterparts, although nymphal development rate and adult survival were reduced [65].

The titers of the three *D*. *citri* bacterial endosymbionts were not different between L8 and L16, two lines with disparate *C*Las acquisition and transmission phenotypes. However, average bacterial titers do not often tell the complete story in *C*Las -*D*. *citri* interactions. A 2015 study by Ramsey and colleagues showed that production of diaphorin and a diaphorin structural variant, two polyketides produced by *“Ca*. P. armatura”, were differentially produced in *C*Las-exposed adult *D*. *citri* [19]. *Wolbachia* may also play a role in *C*Las titer and acquisition variability. The titers of *Wolbachia* were observed to be more variable in guts of *C*Las-exposed *D*. *citri* [14] and positively correlated with *C*Las titer [50, 66]. A small *Wolbachia* protein isolated from *D*. *citri* protein extracts was shown to directly repress the expression of phage lytic cycle genes in *C*Las [24]. Little is known about different *Wolbachia* genotypes that exist in this psyllid and the nature of the phage repressor protein locus in those genotypes. These bacterial symbiont contributions to *C*Las acquisition and transmission are uncharacterized in our isofemale lines, and remain a promising line of future research. We cannot discount variation in the expression of *C*Las effector genes and their interaction with the different isofemale line genotypes as a cause for the variation we observed. At least 16 putative *C*Las effector genes have been identified by bioinformatics analysis, and expression of these genes in plants causes a range of cellular responses, from apoptosis to callose deposition [67]. A comparative analysis of *C*Las gene expression in plants and insects revealed that cohorts of genes are differentially regulated depending on whether the bacterium is in its plant or insect host [68].

Interestingly, the relationship between geographic locations (within Florida) where the parents of our *D*. *citri* isofemale lines were collected and their acquisition and transmission rates seemed variable. For example, the best and poorest acquirer lines (L8 and L16 respectively) were collected from the same location (S1 Table), whereas lines OS1, OS2 and OS3 (all collected from another distant location) were all poor acquirers (Table 1). Thus, in some cases, *D*. *citri* populations (with differences in respect to the acquisition or transmission of *C*Las) seem geographically isolated, while others may be found in the same localities. However, in view of the fact that *D*. *citri* adults can fly for long distances [47], and the lack of real geographic barriers in Florida, this is not surprising. The relationship between geography and vector competence in *D*. *citri* populations may be important epidemiologically in other states, such as California where mountains can provide a significant barrier to gene flow among isolated populations, and requires further investigation.

Although the acquisition and inoculation traits we analyzed here between 2014 and 2016 were relatively stable across several generations, early results from our 2017 tests show that L16, one of the poor vector lines, can acquire at a much higher rate than reported in Table 1 (17 and 46% in two consecutive tests). There are several possible explanations for the recent shift in phenotype, the majority of which have been discussed above to explain the variation in the acquisition and inoculation traits that we observe among the isofemale lines, including host plant effects, *C*Las titer in various parts of the source plant, and titers of the bacterial endosymbionts within the psyllids. *D*. *citri* psyllids are sexually reproducing, and the heterozygosity of these lines for each gene regulating these traits is not known. Ongoing research is focusing on generating sub-lines from these isofemale lines to reduce the heterozygosity at each locus involved. We have also considered new ways to perform the acquisition and inoculation phenotyping, which may reduce the variation we observe. Rather than testing adult progeny from multiple females in each line, we plan to phenotype the progeny from individual females. Experiments to examine variation in transovarial transmission in these lines are also in progress. These new preliminary data for L16 are important point to bring to light now because of the possibility that a poor vector psyllid is not an evolutionary stable genotype. That outcome may complicate the use of poor vectors in any kind of vector replacement management strategy for citrus growers.

The majority of studies from our lab and others investigating the molecular basis of *C*Las transmission have focused on comparing the transcriptome, proteome, and metabolome in insects reared on healthy or *C*Las-infected trees [14, 18, 19, 69, 70]. As *C*Las is unculturable, it is impossible to disentangle the indirect effects of the infected tree on the psyllid’s biology from direct effects of *C*Las in those studies. Consequently, the genes and proteins regulating *C*Las acquisition, transmission and psyllid co-evolution remain largely unknown. Even in the face of the seemingly complex genetic architecture regulating *C*Las acquisition and transmission, these isofemale lines are invaluable tools for future work on understanding the molecular and genetic basis of *C*Las transmission by *D*. *citri*. Research to combine proteomics, transcriptomics and genomics with these valuable insect genetic tools will endeavor to describe the genes and proteins involved in vector-pathogen interactions [32, 36–38, 62, 71–74].

## Materials and methods

### Establishment of isofemale lines

Each isofemale line was started from one female and one male of *D*, *citri*, collected as mentioned above from various Florida locations (S1 Table), released onto a potted, young, healthy (not infected with *C*Las), flushing orange jasmine plant (ca. 6–12 months old) in a cage (Bug Dorm BD44545, 45x45x45 cm, MegaView Science Co., Ltd.) for a period of 1–2 weeks for oviposition. Male and female adults from each pair were then frozen, stored in 95% ethanol and later processed for qPCR, as described below, to determine if they were *C*Las-positive. Only immatures (eggs and nymphs) from *C*Las-negative parents were used to establish the isofemale lines tested here. Samples of 100 adults from each line/colony were also tested by qPCR over 5–6 generations to make sure that the colony was *C*Las-negative before subjecting it to the acquisition or inoculation tests described below. New healthy orange jasmine plants were substituted for the older plants every 4 weeks.

Some isofemale lines (designated GC); however, were established from *C*Las-exposed colonies (#15 and 35) that had been maintained in our laboratory in a growth chamber on *C*Las-infected lemon or citron plants for several years [75]. These two infected colonies were founded using adults from a healthy laboratory colony previously described [76] and were chosen because they were apparently extreme (low and high) in their *C*Las acquisition efficiency. Thus, although both colonies were treated the same over time, a much lower percentage of psyllids from GC-15 usually tested *C*Las-positive by qPCR compared to those from GC-35. Several pairs of female and male adults from each of these two colonies were individually caged on orange jasmine plants for egg laying as described above then tested by qPCR, and only progeny from *C*Las-negative parents were used to establish the GC isofemale lines tested. All colonies were housed in three portable greenhouses with air conditioning (AC) units set to 25°C. However, these AC units malfunctioned a few times during the first year (Oct. 2014- Oct. 2015) and the temperature reached as low as 6.4°C in the winter and as high as 37°C in the summer. For this reason, a few colonies were lost (#K2, K5, L19 and L20) before new and more reliable AC units for these greenhouses were installed.

### Testing isofemale lines for *C*Las acquisition

A group of 50–75 young adults (males and females) from each isofemale line was caged onto a flushing HLB-symptomatic (bud-inoculated) rough lemon plant (*Citrus* x *taitensis* Risso; syn. *Citrus jambhiri* Lush.), pre-confirmed to be qPCR-positive for *C*Las. Rough lemon was used because it produced good HLB symptoms and it produces regular flush, which is necessary for the feeding of psyllid nymphs and egg deposition [1]. The plants were maintained in a growth chamber at 26°C (14 h light, 10 h dark) for about 2 weeks during which the adult females laid eggs, after which the adults were removed. After the eggs hatched and the nymphs developed into adults on this infected plant, at least 50 of these F1 progeny adults (males and females, 1–2 weeks post adult emergence) were collected and tested individually for *C*Las by qPCR. This test was repeated 5 more times in some of the successive generations (S2 Table), with 27 to158 (typically 50–150) adults/test collected and screened for *C*Las by qPCR as described below. In S2 Table, we indicate roughly the no. of generations between the first and each subsequent acquisition test; generation time was estimated to be 3–4 weeks regardless of whether the psyllids were on healthy or diseased plants. From each tested line, 80–138 healthy control psyllid adults (from the healthy isofemale line colony on orange jasmine) were also tested using qPCR along with the infected ones.

### Testing isofemale lines for *C*Las inoculation

Inoculation rates of *C*Las (sometime also referred to here and in the literature as transmission rates) by each isofemale line were assessed using *C*Las-exposed adults (no regard to sex, 1–2 weeks post adult emergence) from nymphs reared on *C*Las-infected rough lemon plants as described above. The ability of these adults to inoculate *C*Las into citrus was determined using an excised leaf assay method described earlier [45]. In each assay repeated in triplicate, a total of 30 healthy young sweet orange leaves, *Citrus* x *aurantium* L., Syn. *Citrus sinensis* (L.) var. Ridge Pineapple, were tested for inoculation by the psyllids, 15 leaves per week over two consecutive weeks. In the first few tests, we caged 6 adults on each leaf for one week, and these adults were transferred to a new leaf for a second week of inoculation. But in the majority of assays, we increased the psyllid numbers in the first week to 10 adults/leaf. Following each inoculation week, each inoculated leaf was washed to remove any psyllid honeydew residues, and incubated in a plastic bag with a moist filter paper (to help maintain freshness) for 7 days at 25°C with 14h light/10h dark before the leaf was frozen (-80°C) then processed for qPCR. At the end of the 2-week inoculation assay, all surviving adults were collected, frozen (-80°C) and processed for qPCR (S2 Table). The average Ct values for the psyllids from this experiment are presented in Table 5, whereas Table 1 presents the average qPCR data from the previous acquisition tests and these experiments.

### Testing an infected laboratory colony (#37) as positive control for isofemale lines

A laboratory colony of *D*. *citri* that has been continuously reared on *C*Las-infected citron for many generations, which originated from a mixed non-isofemale group of laboratory reared psyllids (designated colony #37) was tested by the same qPCR procedure mentioned below as a positive control for the isofemale lines. In this test, two other questions were investigated: a. do males differ from females in their acquisition rates or Ct values, and b. does freezing (-80°C) the *C*Las-exposed psyllids shortly (within an hour) after taking them off infected plants as we did for the isofemale lines, as opposed to clearing their guts by feeding them first for 24 h on healthy citrus leaves, affect percentages that test *C*Las-positive or Ct values. Thus, 60 *D*. *citri* males and females were frozen almost immediately (within 1 h) after taking them off infected pants, and 60 more were fed on excised healthy sweet orange leaves for 24 h, before testing all of them for *C*Las with qPCR. Since evidence shows that *C*Las replicates slowly in psyllid adults [9], we did not use a feeding period longer than 24 h to limit the amount of possible *C*Las multiplication in the psyllids which might confound the results of this test.

### DNA extraction and qPCR of psyllids and citrus leaves

All sample processing and DNA extraction were undertaken in a laminar flow hood. Each psyllid to be tested for qPCR was frozen then stored in 70% ethanol at 4°C until further processing. DNA was extracted from psyllids by using a crude extraction method previously described in detail [45]. For DNA extraction from excised citrus leaves on which *D*. *citri* adults had been fed in the transmission tests, these leaves were washed thoroughly in RNase-Away (Molecular BioProducts, Inc., San Diego, CA), and subsequently rinsed with DI water. Only the midrib of inoculated leaves was processed for qPCR. Each midrib was separated from the leaf blade and chopped into very small pieces with a new sterile razor blade. Each sample was then placed in an individual tube that was subsequently stored in a freezer at -80°C until further processing. DNA was extracted from the leaves using the Nucleo-Spin Plant II kit (Macherey-Nagel, Bethlehem, PA) as described earlier [45].

Extracted DNA from both psyllids and leaves was assayed for the presence of CLas by using the HLBaspr probe/primer set (Li et al. 2006), targeting the 16S DNA of LAS (5’ →3’ sequences: forward TCGAGCGCGTATGCAATACG and reverse GCGTTATCCCGTAGAAAAAGGTAG, probe AGACGGGTGAGTAACGCG with 6-carboxyflourescein reporter dye on the 5’ end and TAMRA quencher on the 3’ end. The HLBaspr primers (Integrated DNA Technologies, Inc. Coralville, IA) were run as a 20-μl reaction using Taqman Fast Universal PCR Master Mix (Applied Biosystems, Foster City, CA), 0.4 mM each of forward and reverse primer, and 500 nM of probe. The temperature program for the HLBaspr primers was 95°C for 5 min followed by 50 cycles of 95 °C for 3 s followed by 60 °C for 30 s. Samples were run in duplicate and rerun in cases when amplification occurred in only one of the two reactions. The primers were run on 96-well skirted plates sealed with TemPlate film (USA Scientific). All qPCR plates contained positive controls and four to six wells of no template and negative controls. All qPCR reactions were run on an ABI 7500 Fast machine and analyzed with 7500 Software version 2.0.1 (Applied Biosystems).

### Characterization of morphological variation between isofemale lines L8 and L16

Adults from these two isofemale lines were collected from citron seedlings. We visualized insects using a bench stereomicroscope (AmScope, SM-1BZ-FRL) and separated them according to sex. The insects were incubated at -20°C for 1 hour prior to the recording of morphological observations. A total of 100 insects from each line were categorized as intermediate, blue, gray or yellow as described previously [77], and the number of insects in each group were counted. This evaluation was performed in three replicates for each isofemale line. To compare among the groups, R was used to perform a two-way ANOVA followed by the TukeyHSD test. Significant p-values reported in Fig 4 were less than 0.01.

### Microbial copy number quantification by PCR

Previous surveys of *D*. *citri* populations found differences in the endosymbiont titer [48]. We tested for this in two of our isofemale lines, L8 and L16, the best and worst acquirer/transmitter lines, respectively. *D*. *citri* samples were frozen and subsequently cryoground (Retsch Mixer Miller MM400), and the total DNA was isolated using the silica-gel membrane-based DNeasy blood and tissue kit (Qiagen). The Applied Biosystems 7900HT instrument was used for PCR analysis. Endosymbiont qPCR was performed using the Fast SYBR Green Master Mix (Life Technologies) and primer sequences from [78]. All qPCR reactions were performed in triplicate and following thermal cycling program: 20 s at 95°C; 40 cycles of (3 s at 95°C; 30 s at 60°C). Absolute quantification of microbial copy number was enabled by comparing Ct values from biological samples to Ct values from standard curves made using serial dilutions of synthetic plasmids containing the qPCR target region.

### Hemocyanin expression in isofemale lines

*D*. *citri* (10 insects per sample and three replicates per each isofemale line) were harvested and subsequently ground using a cryogenic mixer mill (Retsch Mixer Miller MM400). Total RNA was isolated using the RNeasy kit (Qiagen), and the DNA contaminants were removed by treatment of samples with RNase-free DNase I (Thermo Fisher). RNA was quantified using a Nanodrop spectrophotometer (Thermo Fisher). RNA (1 μg) was used for cDNA synthesis using the iScript cDNA Synthesis Kit, and the resulting cDNA was diluted three times and used as template in relative qPCR. Relative qPCR was performed on the Applied Biosystems^™^ QuantStudio^™^ 6 and using the Fast SYBR Green Master Mix (Life Technologies). The hemocyanin primers were Forward: CTCCCCAAGGGATCCAGAGA; Reverse: AAGGACGGTCGAATGGGAAC) to specifically target haemocyanin-1. Alpha tubulin was used as the reference gene for relative quantification with the following primers: a-Tub-F: GCGTCTCTTCGGTTTGACGG and a-Tub-R: CACTTCACCATCTGGTTGGC. The obtained Ct values converted into normalized relative quantities (NRQs) according to the method described in [77] and the yielded values of NRQs were used for statistical analysis. A two-tailed Student’s T test was used to compare the normalized expression levels.

### Statistical analysis

Statistical comparisons among isofemale lines were conducted on percentages of psyllids acquiring *C*Las, on percentage transmission rates of *C*Las by psyllids to leaves, and on titers of *C*Las in psyllids and leaves according to qPCR Ct values. Based on results of the Shapiro-Wilk test for normality within PROC UNIVARIATE in SAS (SAS Institute, 2010) (S4 Table), percentages and Ct values were compared as non-parametric data using the *F*-approximation of the Friedman test [79] and the associated rank sum multiple comparison test with PROC GLIMMIX in SAS. The procedure was used after ranking the data within each sampling date from lowest to highest value using the PROC RANK procedure in SAS. Linear regression/correlation analyses (Pearson’s coefficient) were conducted using PROC CORR in SAS.

## Supporting information

S1 Table Florida locations and dates of collecting *D*. *citri* from *Murraya* trees to establish isofemale lines in the laboratory. (DOCX)

S2 Table *D*. *citri* isofemale lines: Detailed results of *C*Las-acquisition and transmission tests (data summarized in Tables 1 and 5). (DOCX)

S3 Table Results of tests for normality (Shapiro-Wilk procedure) for data presented in Tables 1 and 5. (DOCX)

S1 Fig Mean rates of *C*Las acquisition by 15 isofemale lines of *D*. *citri*; different tests (1–6) performed over several generations (see S2 Table) are shown in different colors to show variability between tests/generations of each line (see also S2 Fig). (TIF)

S2 Fig A scatter diagram of acquisition rates of various isofemale lines over six tests conducted during various generations of *D*. *citri*. No correlation was found between the generation tested and the acquisition rates in all tested lines except for line GC-15-2 (solid line) which had a significantly positive slope between these two variables.
(TIF)
