# Supplementary material for: The quest for a non-vector psyllid: Natural variation in acquisition and transmission of the huanglongbing pathogen ‘Candidatus Liberibacter asiaticus’ by Asian citrus psyllid isofemale lines
Source: PLoS One. 2018 Apr 13;13(4):e0195804. doi: 10.1371/journal.pone.0195804 (PMC5898736; doi:10.1371/journal.pone.0195804)
Supplement: S1 Table — (DOCX) [file pone.0195804.s001.docx]

**S1 Table.** **Florida locations and dates of collecting *D. Citri* from Murraya trees to establish isofemale lines in the laboratory**

| **Isofemale Line** | **Date** | **Location** | **City** | **Coordinates** |
| --- | --- | --- | --- | --- |
| **From the field:**  H2-1, H2-2, H2-3 | 10/28/2015 | Silver Vase Inc. | Homestead | 25°31’14.74”N –80°31’07.10”W |
| K2, K3, K4, K5, K17 | 07/11/2014 | King’s Isle Community | Port St. Lucie | 27°19'2.49"N, –80°23'4.41"W |
| L8, L16, L19, L20 | 08/25/2014 | Laurel Professional Park | Fort Pierce. | 27°24'50.50"N, –80°20'56.65"W |
| OS1, OS2, OS3 | 08/04/2015 | Oyster Shell Rest., 13451 McGregor Blvd #10 | Fort Myers | 26°32’46.00”N, –81°54’47.48”W |
| **From growth chamber colonies:** |  |  |  |  |
| GC 15-2, GC 15-6 |  | ARS-USHRL | Fort Pierce |  |
| GC 35-6, GC 35-7 |  | ARS-USHRL | Fort Pierce |  |
