# Supplementary material for: The quest for a non-vector psyllid: Natural variation in acquisition and transmission of the huanglongbing pathogen ‘Candidatus Liberibacter asiaticus’ by Asian citrus psyllid isofemale lines
Source: PLoS One. 2018 Apr 13;13(4):e0195804. doi: 10.1371/journal.pone.0195804 (PMC5898736; doi:10.1371/journal.pone.0195804)
Supplement: S2 Table — (DOCX) [file pone.0195804.s002.docx]

**S2 Table. *D. citri* isofemale lines: Detailed results of CLas-acquisition and transmission tests (data summarized in Tables 1 and 4)**

| **Line^1^** | **Date** | **Test**  **No.** | **Generation^2^** | **CLas-**  **Acquisition^3^** | | **CLas inoculation/**  **Transmission^4^** | | |
| --- | --- | --- | --- | --- | --- | --- | --- | --- |
|  |  |  |  | **No** | **%** | **Wk1** | **Wk2** | **Total** |
| **L8** | 12/01/14*  08/11/15  10/01/15  11/17/15  06/01/16*  06/01/16* | 1  2  3  4  5  6 | 1  11  13  15  24  24 | 14/60  23/50  18/37  18/50  26/135  29/155 | 23.33  46.00  48.65  36.00  19.26  18.71 | 2/15  3/15  7/15 | 4/15  2/15  4/15 | 6/30  5/30  11/30 |
|  |  |  | Total  % | 128/487  26.28 |  | 12/45  26.7 | 10/45  22.2 | 22/9024.4 |
| **H2-1** | 02/26/16  03/24/16  05/10/16  05/31/16*  05/31/16*  05/31/16* | 1  2  3  4  5  6 | 1  2  5  6  6  6 | 25/50  15/50  9/48  31/130  37/135  42/130 | 50.0  30.0  18.75  23.85  27.41  32.31 | 2/15  4/15  4/15 | 7/15  3/15  5/15 | 9/30  7/30  9/30 |
|  |  |  | Total  % | 159/543  29.28 |  | 10/45  22.2 | 15/45  33.3 | 25/90  27.8 |
| **K4** | 02/19/15  03/04/15*  01/04/16  01/26/16  09/15/16*  10/24/16* | 1  22  3  4  5  6 | 1  2  14  15  25  27 | 12/43  16/70  9/50  5/50  52/105  60/142 | 27.91  22.86  18.00  10.00  49.52  42.25 | 1/15  9/15  7/15 | 0/15  0/15  0/15 | 1/30  9/30  7/30 |
|  |  |  | Total  % | 154/460  33.48 |  | 17/45  37.8 | 0/45  0.0 | 17/90  18.9 |
| **GC35-7** | 01/04/16  01/29/16  02/26/16  04/08/16*  04/08/16*  04/08/16* | 1  2  3  4  5  6 | 1  2  3  5  5  5 | 9/50  14/50  23/50  15/123  27/133  18/127 | 18.0  28.0  46.0  12.20  20.30  14.17 | 0/15  2/15  0/15 | 0/15  0/15  2/15 | 0/30  2/30  2/30 |
|  |  |  | Total  % | 106/533  19.89 |  | 2/45  4.4 | 2/45  4.4 | 4/90  4.4 |
| **H2-2** | 03/24/16  05/10/16  06/30/16  08/04/16  08/04/16  08/04/16 | 1  2  3  4  5  6 | 1  3  5  7  7  7 | 17/50  2/50  14/50  18/134  24/132  25/141 | 34.0  4.0  28.0  13.43  18.18  17.73 | 1/15  0/15  0/15 | 1/15  0/15  0/15 | 2/30  0/30  0/30 |
|  |  |  | Total  % | 100/557  17.95 |  | 1/45  2.2 | 1/45  2.2 | 2/90  2.2 |
| **GC15-6** | 01/04/16  02/26/16  03/24/16  04/28/16*  04/28/16*  04/28/16* | 1  2  3  4  5  6 | 1  3  4  6  6  6 | 5/27  23/50  7/50  20/136  12/151  22/158 | 18.52  46.0  14.0  14.71  7.95  13.92 | 2/15  2/15  2/15 | 2/15  0/15  1/15 | 4/30  2/30  3/30 |
|  |  |  | Total  % | 89/572  15.56 |  | 6/45  13.3 | 3/45  6.7 | 9/90  10.0 |
| **GC15-2** | 08/17/16  09/16/16  12/01/16  12/19/16  12/19/16  12/19/16 | 1  2  3  4  5  6 | 1  2  5  6  6  6 | 3/50  8/50  9/50  35/127  25/130  32/128 | 6.00  16.0  18.0  27.56  19.23  25.00 | 4/15  3/15  2/15 | 2/15  2/15  0/15 | 6/30  5/30  2/30 |
|  |  |  | Total  % | 112/535  20.93 |  | 9/45  20.0 | 4/45  8.9 | 13/90  14.4 |
| **K17** | 04/30/15*  11/17/15  01/04/16  01/29/16  02/26/16*  02/26/16* | 1  2  3  4  5  6 | 1  10  12  13  14  14 | 25/135  4/50  4/51  6/49  39/140  32/118 | 18.52  8.0  7.84  12.24  27.86  27.12 | 1/15  0/15  0/15 | 1/15  1/15  0/15 | 2/30  1/30  0/30 |
|  |  |  | Total  % | 110/543  20.26 |  | 1/45  2.2 | 2/45  4.4 | 3/90  3.3 |
| **GC35-6** | 07/22/16  09/16/16  11/05/16*  11/05/16*  11/05/16*  12/01/16 | 1  2  3  4  5  6 | 1  3  5  5  5  6 | 1/50  6/50  2/50  31/131  37/130  32/119 | 2.0  12.0  4.0  23.66  28.46  26.89 | 6/15  4/15  5/15 | 4/15  2/15  2/15 | 10/30  6/30  7/30 |
|  |  |  | Total  % | 109/530  20.57 |  | 15/45  33.3 | 8/45  17.8 | 23/90  25.6 |
| **K3** | 07/09/15*  08/11/15  11/17/15  01/04/16  09/15/16*  10/24/16* | 1  2  3  4  5  6 | 1  2  6  8  18  19 | 24/120  22/64  2/51  2/50  7/108  13/131 | 20.0  34.38  3.92  4.0  6.48  9.92 | 0/15  4/15  2/15 | 0/15  4/15  0/15 | 0/30  8/30  2/30 |
|  |  |  | Total  % | 70/524  13.36 |  | 6/45  13.3 | 4/45  8.9 | 10/90  11.1 |
| **H2-3** | 02/26/16  03/24/16  05/10/16  06/02/16*  06/02/16*  06/02/16* | 1  2  3  4  5  6 | 1  2  4  5  5  5 | 4/51  16/50  0/50  11/101  7/113  6/138 | 7.84  32.0  0.0  10.89  6.19  4.35 | 1/11  1/13  0/15 | 0/15  2/15  0/15 | 1/26  3/28  0/30 |
|  |  |  | Total  % | 44/503  8.75 |  | 2/39  5.13 | 2/45  4.4 | 4/84  4.8 |
| **OS1** | 06/23/16  07/19/16  08/17/16  08/30/16*  08/30/16*  08/30/16* | 1  2  3  4  5  6 | 1  2  3  4  4  4 | 3/50  9/50  3/50  10/137  11/136  16/123 | 6.0  18.0  6.0  7.30  8.09  13.01 | 1/15  1/15  0/15 | 0/15  0/15  0/15 | 1/30  1/30  0/30 |
|  |  |  | Total  % | 52/546  9.52 |  | 2/45  4.4 | 0/45  0.0 | 2/90  2.2 |
| **OS2** | 05/10/16  09/16/16  12/01/16  12/15/16*  12/15/16*  12/15/16* | 1  2  3  4  5  6 | 1  6  9  10  10  10 | 0/30  4/50  11/50  15/129  6/132  3/114 | 0.0  8.0  22.0  11.63  4.55  2.63 | 0/15  1/15  2/15 | 0/15  0/15  0/15 | 0/30  1/30  2/30 |
|  |  |  | Total  % | 39/505  7.72 |  | 3/45  6.7 | 0/45  0.0 | 3/90  3.3 |
| **OS3** | 01/04/16  01/26/16  02/26/16  03/15/16*  03/28/16*  03/28/16* | 1  2  3  4  5  6 | 1  2  3  4  5  5 | 2/40  5/45  5/50  8/146  3/147  4/156 | 5.0  11.1  10.0  5.48  2.04  2.56 | 1/15  0/15  0/15 | 0/15  0/15  0/15 | 1/30  0/30  0/30 |
|  |  |  | Total  % | 27/584  4.62 |  | 1/45  2.2 | 0/45  0.0 | 1/90  1.1 |
| **L16** | 04/06/15  04/20/15*  08/11/15  11/17/15  07/18/16*  07/18/16* | 1  2  3  4  5  6 | 1  2  6  10  20  20 | 5/49  8/112  0/30  1/50  4/138  11/144 | 10.20  7.14  0.0  2.0  2.9  7.64 | 0/15  0/15  0/15 | 0/15  0/15  0/15 | 0/30  0/30  0/30 |
|  |  |  | Total  % | 29/523  5.54 |  | 0/45  0.0 | 0/45  0.0 | 0/90  0.0 |

**^1^** Lines arranged descendingly according to mean CLas-acquisition rate.

**^2^** Estimated no. of generations between the first and each subsequent acquisition test; generation time was estimated to be 3-4 weeks regardless of whether the psyllids were on healthy or diseased plants.

**^3^** Six acquisition tests/line with 27-158 (typically 50-150) psyllid adults/test; tested psyllids had been reared as nymphs on CLas-infected lemon plants for one or more generations.

**^4^** Three transmission tests/line, 15 excised leaves/test/week for 2 consecutive weeks (90 leaves total), with 6-10 psyllids/leaf/week; inoculating psyllids had been reared as nymphs on CLas-infected rough lemon plants for one or more generations.

*On these dates, psyllids used for CLas inoculation into excised leaves in the transmission tests were tested by qPCR for CLas acquisition.
