# Supplementary material for: The quest for a non-vector psyllid: Natural variation in acquisition and transmission of the huanglongbing pathogen ‘Candidatus Liberibacter asiaticus’ by Asian citrus psyllid isofemale lines
Source: PLoS One. 2018 Apr 13;13(4):e0195804. doi: 10.1371/journal.pone.0195804 (PMC5898736; doi:10.1371/journal.pone.0195804)
Supplement: S3 Table — (DOCX) [file pone.0195804.s003.docx]

| **S3 Table.** Results of tests for Normality (Shapiro-Wilk procedure) for data presented in Tables 1 and 5. | | | | |
| --- | --- | --- | --- | --- |
| Data variable compared among lines | Table | N | W statistic | *P* value |
| Percentage of psyllids acquiring CLas | 1 | 90 | 0.916 | <0.0001 |
| qPCR Ct value of CLas-infected psyllids | 1 | 87 | 0.965 | 0.020 |
| Percent transmission rates to citrus leaves | 3 | 90 | 0.776 | <0.0001 |
| qPCR Ct value of CLas-infected leaves | 3 | 49 | 0.691 | <0.0001 |
| Percentage of CLas-infected psyllids | 3 | 90 | 0.924 | <0.0001 |
| qPCR Ct value of CLas-infected psyllids | 3 | 43 | 0.944 | 0.036 |
