# Supplementary figures and images for: The quest for a non-vector psyllid: Natural variation in acquisition and transmission of the huanglongbing pathogen ‘Candidatus Liberibacter asiaticus’ by Asian citrus psyllid isofemale lines
Source: PLoS One. 2018 Apr 13;13(4):e0195804. doi: 10.1371/journal.pone.0195804 (PMC5898736; doi:10.1371/journal.pone.0195804)

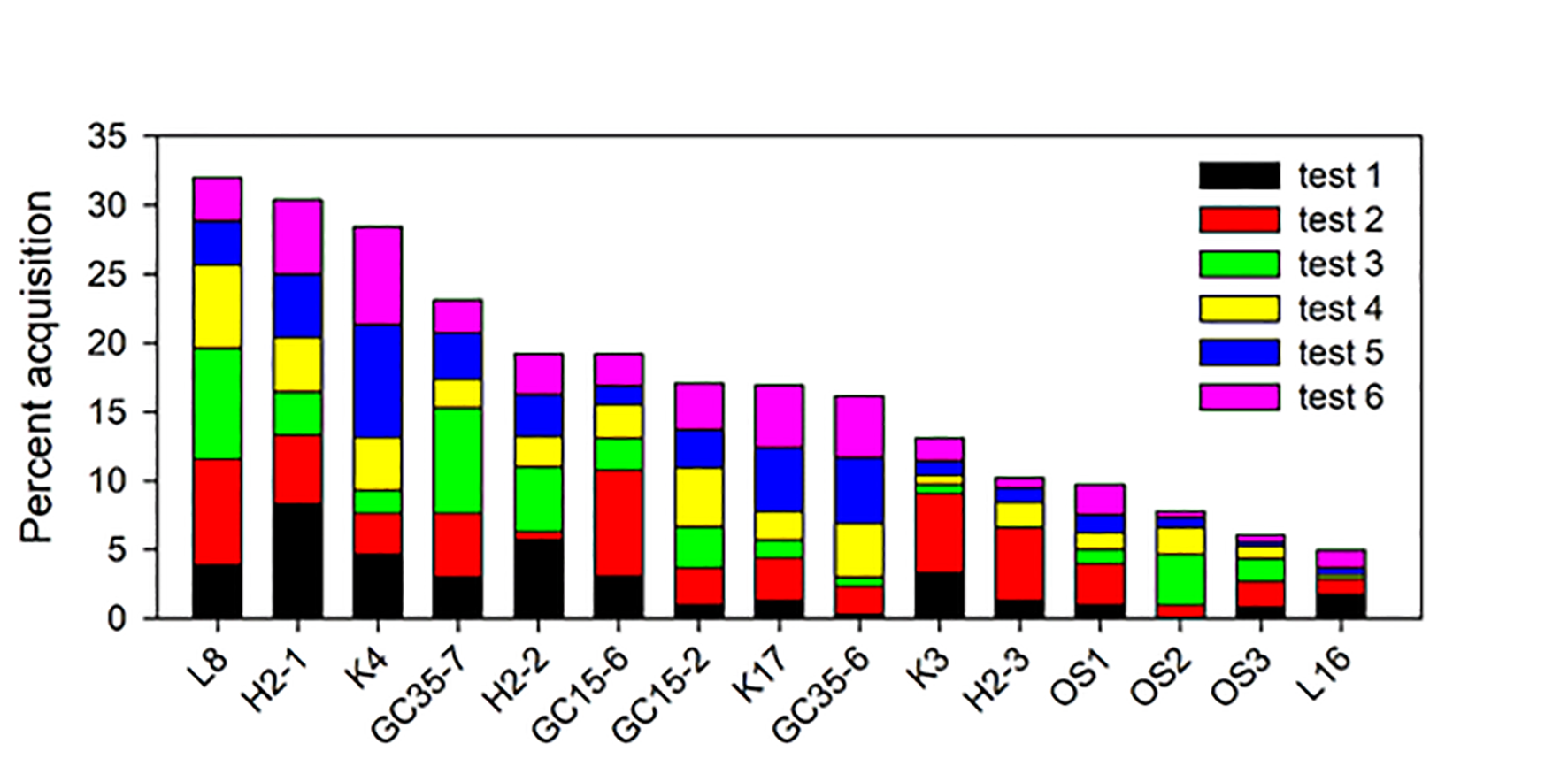

Supplement: S1 Fig — (TIF) [file pone.0195804.s004.tif]

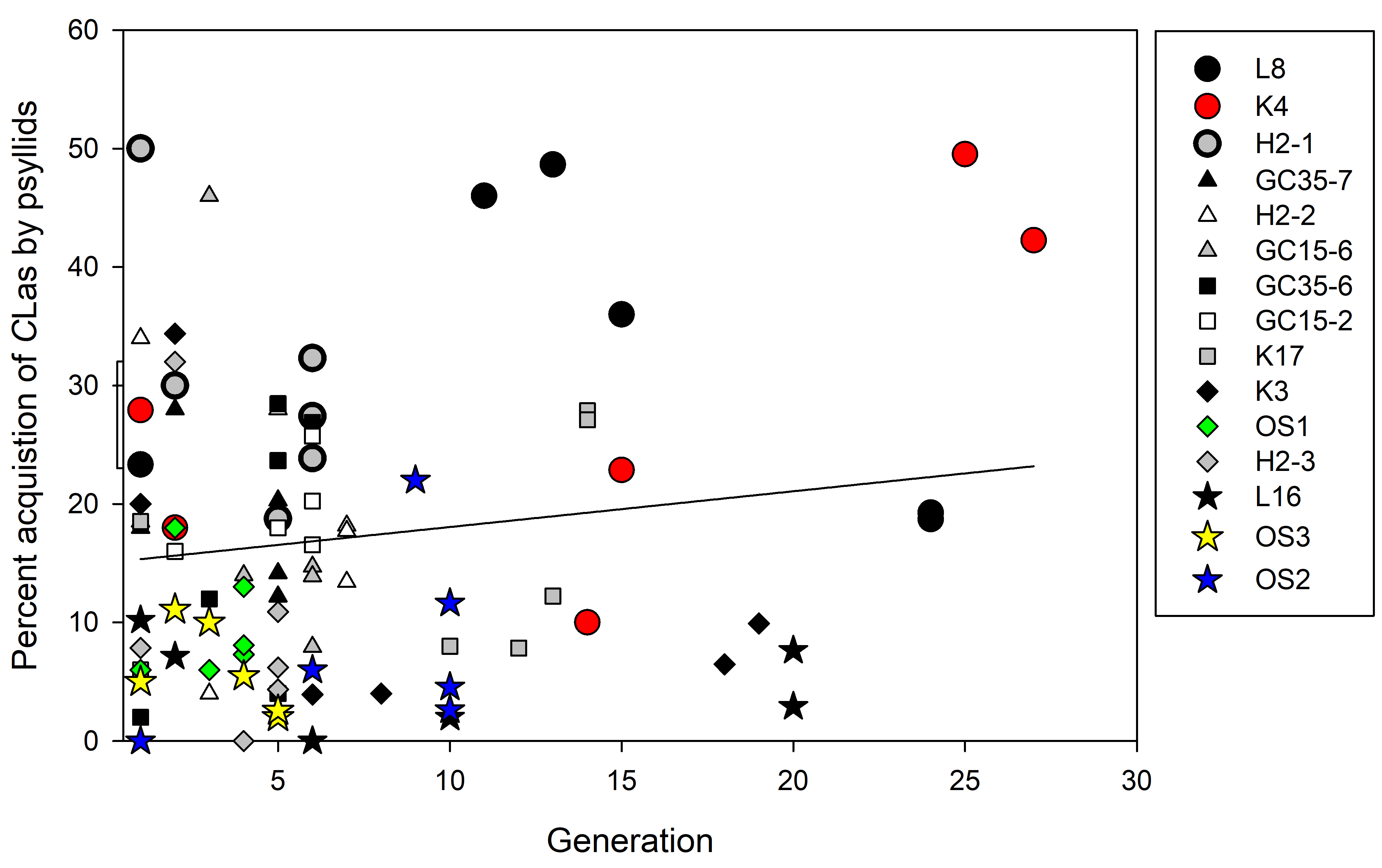

Supplement: S2 Fig — No correlation was found between the generation tested and the acquisition rates in all tested lines except for line GC-15-2 (solid line) which had a significantly positive slope between these two variables. (TIF) [file pone.0195804.s005.tif]
